# dNLR, NLR, and NMLR as differential markers for assessing severity and sepsis in emphysematous pyelonephritis

**DOI:** 10.3389/fmed.2026.1831260

**Published:** 2026-07-15

**Authors:** Maidina Aisihaer, Asimujiang Abula, Guanglu Song

**Affiliations:** Department of Urology, First Affiliated Hospital of Xinjiang Medical University, Urumqi, China

**Keywords:** biomarkers, complete blood count, emphysematous pyelonephritis, inflammation, sepsis

## Abstract

**Purpose:**

This study aims to evaluate the association between blood count-derived inflammatory markers and the severity of radiographic disease, as well as the presence of sepsis in patients with Emphysematous pyelonephritis.

**Methods:**

This retrospective cohort study included 47 adult patients diagnosed with EPN. Anatomical severity was assessed using Huang’s grading system based on admission CT scans. Sepsis diagnosis followed the Sepsis-3.0 criteria. CBC-derived indices were calculated from initial admission blood test results. Intergroup comparisons, effect size analysis, receiver operating characteristic curves, and correlation analyses were performed.

**Results:**

The derived neutrophil-to-lymphocyte ratio (dNLR) had a statistically significant association with Huang’s CT classification (*H* = 9.555, *p* = 0.023), with median values increasing with severity grade. In sepsis, both NLR and NMLR levels were considerably higher (*p* = 0.041 and *p* = 0.033, respectively). NMLR had the largest effect size (*r* = 0.45) and the best discriminatory performance (AUC = 0.683, 95% CI: 0.520–0.846). Internal correlation analysis found that NLR and NMLR were highly correlated, while dNLR was weakly correlated with them, suggesting distinct dimensions of information. However, none CBC-derived inflammatory markers were related to long-term unfavorable outcomes (death or permanent dialysis), which were robustly predicted by traditional renal function markers (creatinine and cystatin C; all *p* < 0.05).

**Conclusion:**

dNLR, NLR, and NMLR are readily obtainable CBC-derived indicators that show distinct clinical associations in EPN. dNLR correlates with anatomical disease severity, while NLR and NMLR are superior indicators for identifying concomitant sepsis. These inexpensive markers offer practical, complementary tools for acute-phase risk stratification, aiding in the timely recognition of severe disease and systemic complications.

## Introduction

Emphysematous pyelonephritis (EPN) is a rare, necrotizing infection of the renal parenchyma and perirenal area, characterized by the infection of the collecting system, renal parenchyma, or perirenal tissues, as well as the production of infectious gases, a signature feature of EPN (1). It occurs mainly in female patients and is potentially fatal, with a mortality rate of 40–90%. EPN is caused by a urinary tract infection, which can quickly spread to the renal parenchyma, causing multiple organ failure, infectious shock, sepsis, and even death (2). Diabetes mellitus (DM) is the most common comorbidity among EPN patients. The condition’s pathophysiology involves hyperglycemia, gas-forming infections, and impaired immunity, with common symptoms including back pain, fever, vomiting and shock (3).

The primary diagnostic method for EPN is imaging. X-rays may show gas in the kidney. Ultrasound reveals bright gas shadows in the renal parenchyma, calyces, or renal pelvis (4). Computed tomography (CT) is the definitive imaging modality for EPN, enabling accurate diagnosis and anatomical staging (5). In 1996, Wan et al. (6) used CT to classify EPN. Category I featured renal parenchymal damage without substantial effusion or striated gas, and a 70% mortality rate. Category II showed effusions with gas in the kidney, perirenal area, or collecting system, with a 20% mortality, indicating a better prognosis. Afterwards, Huang et al. (7) categorized EPN into four CT-based grades, significantly aiding patient management and prognosis. Class 1 involves gas exclusively in the collecting system; Class 2 involves gas in the renal parenchyma without perirenal extension. Class 3A includes perirenal gas/abscesses, and Class 3B, pararenal gas/abscesses. Class 4 indicates bilateral or isolated kidney disease. However, while imaging excels at identifying anatomical severity, it cannot directly quantify the concurrent systemic inflammatory-immune dysregulation, which is the fundamental pathological mechanism causing life-threatening sepsis, which continues to be the leading cause of death in EPN. It is worth noting that the complete blood count (CBC) is a standard laboratory assessment that includes white blood cells, red blood cells, and platelets (8). The assessment of certain inflammatory markers from complete blood counts, including the neutrophil-to-lymphocyte ratio (NLR), derived neutrophil-to-lymphocyte ratio (dNLR), monocyte-to-lymphocyte ratio (MLR), neutrophil-monocyte to lymphocyte ratio (NMLR), systemic inflammatory response index (SIRI), and systemic immune-inflammation index (SII), is vital for the effective diagnosis and management of diverse medical issues (9–11). Several studies conducted outside of the urological literature used the neutrophil-to-lymphocyte ratio (NLR) to predict a patient’s likelihood of developing urosepsis following PCNL (12, 13). Within the field of urology, these metrics have been used to predict SIRS criteria and the risk of urosepsis in individuals undergoing percutaneous nephrolithotomy (12, 14). Consistently, augmented levels of both indices demonstrate a strong association with individuals fulfilling the SIRS diagnostic parameters and subsequently developing sepsis (15, 16). However, the interrelation between CBC-derived inflammatory indicators and the incidence of adverse events in EPN patients remains unexplored.

Current management of EPN relies on imaging for anatomical staging. However, it lacks dynamic biomarkers that reflect systemic inflammatory-immune axis dysregulation-the main driver of sepsis and organ failure. CBC-derived indices quantify both neutrophil-driven inflammation and lymphocyte-mediated immune competence. These measures offer a unique window into the disease’s pathophysiological state. However, their specific associations with anatomical severity and clinical outcomes in EPN remain unclear. Therefore, this retrospective study aimed to systematically evaluate the associations of multiple CBC-derived inflammatory indices (NLR, dNLR, MLR, NMLR, SII, SIRI) with radiographic disease severity (Huang’s classification) and the presence of sepsis in patients with EPN.

## Materials and methods

### Participants and variables

This retrospective cohort study was conducted at *the First Affiliated Hospital of Xinjiang Medical University*. The medical records of 47 adult patients (aged 18 years or older) diagnosed with emphysematous pyelonephritis (EPN) between January 2016 and June 2023 were reviewed. The diagnosis was established based on computed tomography (CT) findings, which demonstrated the presence of gas within the renal parenchyma, collecting system, and/or perirenal spaces. Exclusion criteria for participants involved the presence of a documented fistula connecting the urinary and gastrointestinal systems, a history of recent urogenital instrumentation or trauma, or inadequate clinical or laboratory information. The baseline characteristics recorded for each subject encompassed age, gender, body mass index, and pre-existing medical conditions, notably diabetes mellitus. Admission laboratory measurements comprised a full blood count, serum creatinine levels, blood glucose concentrations, and any relevant inflammatory indices subsequently computed. The primary CBC-derived inflammatory indices for association analyses were calculated using the results from the first complete blood count obtained at hospital admission.

To elucidate the role of systemic inflammation in disease progression, this study defined key outcomes and variables. The diagnosis of sepsis strictly followed the Sepsis 3.0 criteria, based on acute changes in the Sequential Organ Failure Assessment (SOFA) score. Anatomical severity was graded using Huang’s classification method based on admission CT scans. This study calculated various blood cell count-derived inflammatory markers, including the neutrophil-to-lymphocyte ratio (NLR), derived NLR (dNLR), monocyte-to-lymphocyte ratio (MLR), neutrophil-monocyte-to-lymphocyte ratio (NMLR), systemic immune inflammation index (SII), and systemic inflammatory response index (SIRI), aiming to comprehensively quantify neutrophil-dominated inflammation and lymphocyte-represented immune status.

Fasting venous blood samples were collected from all participants upon admission for the measurement of their leukocyte, neutrophil, lymphocyte, and monocyte counts (expressed as 10^9^ cells/L). The following inflammatory indices were calculated using the following formulae:

- NLR = neutrophil count/lymphocyte count
- dNLR = neutrophil count/(white blood cell count – lymphocyte count)
- MLR = monocyte count/lymphocyte count
- NMLR = (neutrophil count + monocyte count)/lymphocyte count
- SII = (platelet count × neutrophil count)/lymphocyte count
- SIRI = (neutrophil count × monocyte count)/lymphocyte count

### Statistical analysis

All statistical analyses were conducted using patients as independent units of analysis. Normality of continuous variables was assessed using the Shapiro–Wilk test. As most variables were not normally distributed, data are described using the median with interquartile range (IQR) [*M* (P25, P75)]. Categorical variables were expressed as frequency and percentage [*n* (%)].

Group comparisons were performed as follows: For comparisons between two groups, continuous variables were analyzed using the Mann–Whitney *U* test, and categorical variables using the Chi-square test or Fisher’s exact test, as appropriate. For comparisons across multiple groups (e.g., Huang’s classification grades), the Kruskal–Wallis *H* test was used for continuous variables, and the Chi-square test for categorical variables.

To quantify the magnitude of observed differences or associations, effect sizes were calculated for significant non-parametric tests and reported as rank-biserial correlation coefficients (*r*), where |*r*| ≥ 0.1, 0.3, and 0.5 typically represents small, medium, and large effect sizes, respectively, where the absolute value indicates the strength of association.

To rigorously assess the discriminatory ability of specific indicators, such as NLR and NMLR, in differentiating sepsis status, receiver operating characteristic (ROC) curves were constructed. The area under the curve (AUC), optimal cutoff values, sensitivity, specificity, positive predictive value (PPV), and negative predictive value (NPV) were calculated. To examine the interrelationships among the six CBC-derived inflammatory indices, we calculated Spearman’s rank correlation coefficients between each marker pair. The resulting correlation matrix was visualized as a heatmap. All statistical tests were two-tailed, with *p* < 0.05 indicating statistically significant differences. Statistical analyses were performed using SPSS (version 25.0). Figures, including the correlation heatmap, were generated using R (version 4.0.3).

No artificial intelligence (AI) tools, including large language models (LLMs) such as ChatGPT, were used in the conception, writing, or data analysis of this manuscript. All work was performed by the human authors.

## Result

### Baseline characteristics of the study cohort

The baseline characteristics of the 47 patients with EPN are summarized in Table 1. The cohort’s median age was 60.5 years (interquartile range: 48.5–70.0) and its median BMI was 22.0 (20.0–27.8). The majority of the participants were women (70.2%). Diabetes was a common comorbidity, affecting 74.5% of patients. According to Huang’s CT classification, more than half of the patients (51.1%) were in class I, followed by class III (40.4%), with classes II and IV being less common (2.1 and 6.4%, respectively). Pancreatic involvement was slightly more common on the right side (53.2%) than on the left (46.8%). During the stay, 20 individuals (42.6%) satisfied the criteria for sepsis.

**Table 1:** Baseline characteristics of study participants (*n* = 47).

Variable	Total
Gender	
Male	14 (29.8%)
Female	33 (70.2%)
Age	60.5 (48.5, 70.0)
Combined diabetes	35 (74.5%)
Huang’s classification	
I	24 (51.1%)
II	1 (2.1%)
III	19 (40.4%)
IV	3 (6.4%)
Sepsis	20 (42.6%)
BMI	22 (20.0, 27.8)
Left side	22 (46.8%)
Right side	25 (53.2%)

Data are presented as number (%) for categorical variables and median (25th, 75th percentiles) for continuous variables. Huang’s classification refers to the CT-based staging system for acute pancreatitis. Sepsis was diagnosed according to the Sepsis-3.0 criteria. BMI, body mass index.

### Associations between CBC-derived indicators and imaging severity

**Figure 1:**
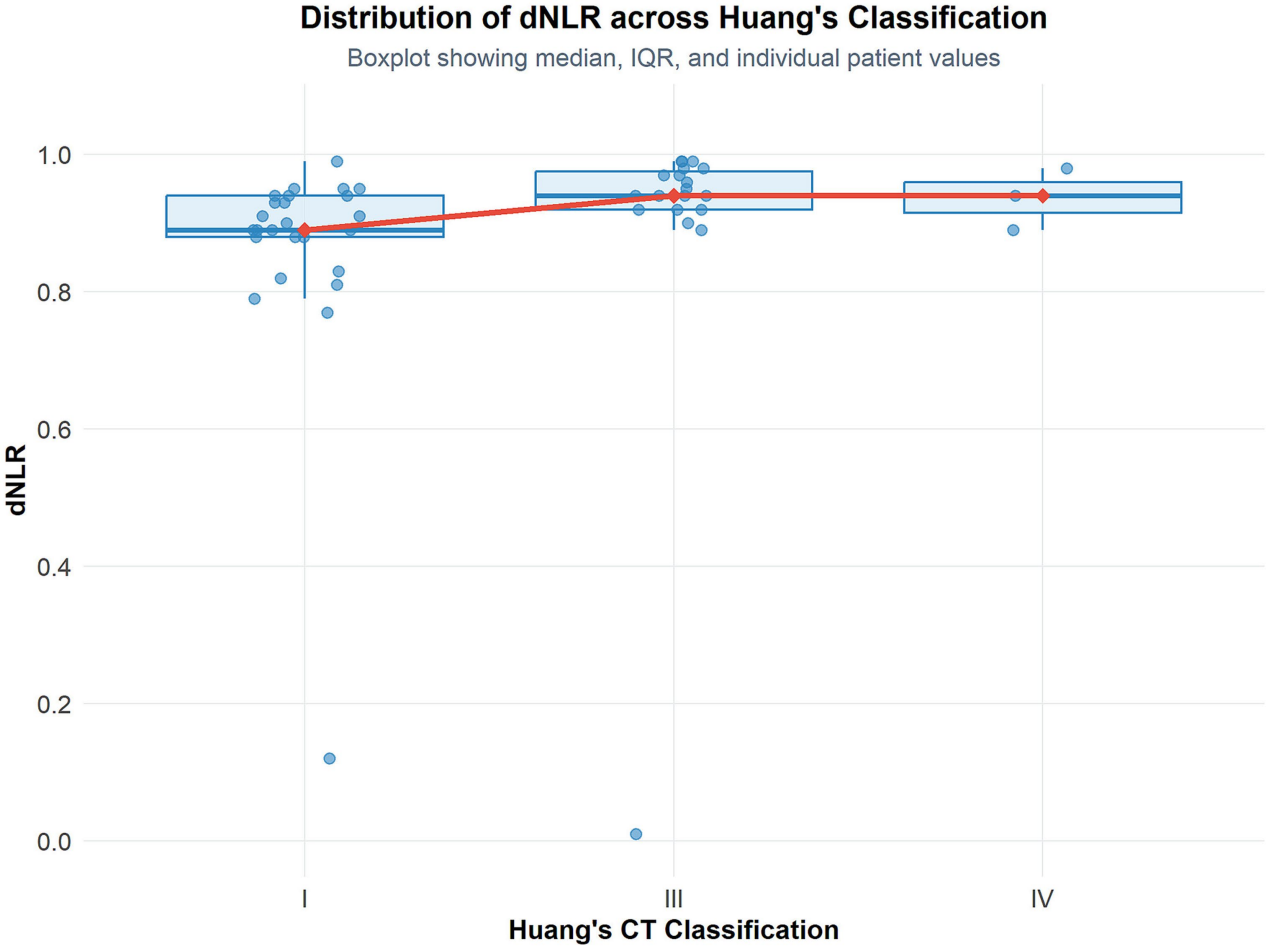
Boxplot showing the distribution of derived neutrophil-to-lymphocyte ratio (dNLR) across Huang’s CT classification groups. The box represents the interquartile range (IQR, 25th to 75th percentile), the horizontal line inside the box denotes the median, and the whiskers extend to the minimum and maximum values within 1.5 × IQR. Individual data points are overlaid. Group II (*n* = 1) is shown as a single point. The Kruskal–Wallis test indicated a statistically significant difference among groups (*p* = 0.023).

The relationships between CBC-derived inflammatory indicators and disease severity according to Huang’s CT classification are summarized in Table 2. A statistically significant difference among the groups was observed for the dNLR (*H* = 9.555, *p* = 0.023). The median dNLR exhibited a progressive increase from class I [0.90 (0.83, 0.94)] to class III [0.94 (0.92, 0.98)] and class IV [0.94 (0.92, 0.97)]. As shown in Figure 1, dNLR increased progressively over the severity spectrum, with median (IQR) values going from 0.90 (0.83–0.94) in class I to 0.94 (0.92–0.98) in class III and 0.94 (0.92–0.97) in class IV. Notably, while the median dNLR was equal in classes III and IV, the distribution patterns within these groups varied significantly (Figure 1). This progressive elevation suggests a potential link between neutrophil-dominated systemic inflammation and advancing anatomical severity in EPN. Other neutrophil-centric indices (NLR, NMLR, SII) showed the highest median values in class III patients, but these trends were not statistically significant (all *p* > 0.05, Table 2). There were no clear relationships detected for MLR or SIRI.

**Table 2:** Comparison of CBC-derived inflammatory indicators across Huang’s CT classification groups.

Variable	Total (*n* = 47)	I (*n* = 24)	II (*n* = 1)	III (*n* = 19)	IV (*n* = 3)	*H*	*p*
NLR	9.79 (4.98, 19.24)	7.29 (2.36, 9.92)	–	16.11 (7.06, 34.73)	11.35 (7.25, 13.27)	6.915	0.075
dNLR	0.93 (0.89, 0.96)	0.90 (0.83, 0.94)	–	0.94 (0.92, 0.98)	0.94 (0.92, 0.97)	9.555	0.023
MLR	0.57 (0.27, 0.97)	0.44 (0.23, 0.93)	–	0.67 (0.49, 1.12)	0.74 (0.50, 0.83)	2.693	0.441
NMLR	9.88 (5.25, 20.13)	8.17 (2.59, 10.55)	–	17.12 (7.611, 36.46)	12.08 (7.75, 14.10)	6.868	0.076
SIRI	5.44 (1.53, 12.39)	3.44 (0.88, 9.82)	–	5.72 (2.86, 16.84)	10.89 (6.31, 11.45)	2.604	0.457
SII	1,956.39 (931.03, 3,438.14)	1,389.42 (780.55, 2,722.66)	–	3,070.83 (1,886.42, 6,232.00)	1,064.68 (994.96, 1,360.61)	6.413	0.093

Data are presented as median (25th, 75th percentiles). Group comparisons were performed using the Kruskal–Wallis test (reported as *H* statistic and *p* value). The Group II (*n* = 1) was excluded from statistical comparisons due to an insufficient sample size, data for this group are shown as “–”.

### Associations between CBC-derived indicators and sepsis

**Figure 2:**
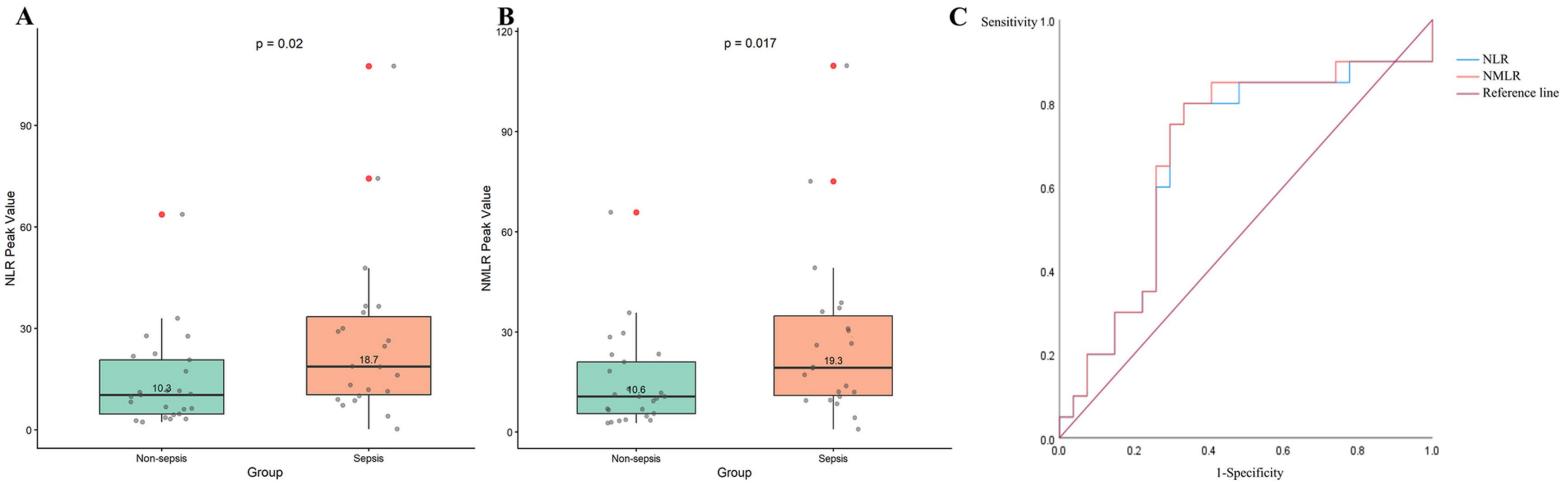
Boxplots comparing peak values of NLR and NMLR between non-sepsis and sepsis groups and ROC curve. **(A)** Comparison of peak NLR levels: non-sepsis group (*n* = 25) vs. sepsis group (*n* = 22), *p* = 0.02. **(B)** Comparison of peak NMLR levels: non-sepsis group (*n* = 25) vs. sepsis group (*n* = 22), *p* = 0.017. Boxes represent interquartile ranges (25th to 75th percentiles), with the horizontal line within the box denoting the median. Whiskers extend to the minimum and maximum values within 1.5 times the interquartile range. Outliers are indicated by external points. **(C)** The area under the curve (AUC) for NLR was 0.676 (95% CI: 0.512–0.840), while the AUC for NMLR was 0.683 (95% CI: 0.520–0.846). The reference line (dashed line) represents no discriminatory ability (AUC = 0.5).

To assess the association between CBC-derived inflammatory markers and the occurrence of sepsis, we first compared the differences in marker levels between the non-sepsis group (*n* = 25) and the sepsis group (*n* = 22) (Table 3). Patients in the sepsis group exhibited significantly higher levels of both the NLR and the NMLR compared to the non-sepsis group (NLR: 12.58 [8.91, 28.43] vs. 6.70 [3.18, 17.33], *p* = 0.041; NMLR: 12.94 [9.71, 29.84] vs. 6.95 [3.36, 18.26], *p* = 0.033). Effect size analysis further revealed that the intergroup difference in NMLR exhibited the largest effect size (*r* = 0.45), indicating a moderate to strong correlation with sepsis status (Table 3). Differences in peak distribution of NLR and NMLR between groups were visually represented via box plots (Figure 2).

**Table 3:** Comparison of CBC-derived inflammatory indicators between sepsis and non-sepsis groups and their effect sizes.

Variable	Non-sepsis (*n* = 25)	Sepsis (*n* = 22)	*U*	*p*	Effect size (*r*)
NLR	6.70 (3.18,17.33)	12.58 (8.91,28.43)	4.178	0.041	0.43
dNLR	0.91 (0.88,0.95)	0.94 (0.91,0.98)	3.249	0.071	0.37
MLR	0.46 (0.24,0.93)	0.71 (0.53,1.03)	2.110	0.146	0.25
NMLR	6.95 (3.36,18.26)	12.94 (9.71,29.84)	4.538	0.033	0.45
SIRI	3.52 (0.88,13.60)	7.95 (2.70,12.51)	0.896	0.344	0.12
SII	1,958 (961.51, 3,661.39)	1,563.10 (891.65, 4,198.22)	0.204	0.651	0.06

Data are presented as median (25th percentile, 75th percentile). Intergroup comparisons were performed using the Mann–Whitney *U* test, with both *U* values and *p* values reported. Effect size (r) was calculated based on test results (formula: *r* = *Z*/ √ *N*), with magnitude defined as: small (0.1), moderate (0.3), large (0.5).

We further evaluated the discriminatory performance of NLR and NMLR for sepsis using receiver operating characteristic (ROC) curves. ROC analysis (Figure 2) showed that the area under the curve (AUC) for NLR and NMLR was 0.676 (95% CI: 0.512–0.840) and 0.683 (95% CI: 0.520–0.846), respectively. At optimal diagnostic thresholds (NLR: 0.467; NMLR: 0.454), NLR demonstrated sensitivity of 80.0% and specificity of 66.7%, while NMLR showed sensitivity of 75.0% and specificity of 70.4%. Detailed diagnostic performance metrics are presented in Table 4. In summary, NLR and NMLR were significantly elevated in patients with sepsis, with NMLR exhibiting the largest between-group effect size. ROC analysis indicated that both markers demonstrated moderate discriminatory ability for sepsis.

**Table 4:** ROC curve analysis of NLR and NMLR for predicting sepsis.

Diagnostic criteria	Optimal diagnostic threshold	AUC (95%CI)	Sensitivity	Specificity	PPV	NPV	LR+	LR−
NLR	0.467	0.676 (0.512–0.840)	0.800	0.667	0.640	0.818	2.400	0.300
NMLR	0.454	0.683 (0.520–0.846)	0.750	0.704	0.652	0.792	2.531	0.355

AUC, area under the curve; CI, confidence interval; PPV, positive predictive value; NPV, negative predictive value; LR+, positive likelihood ratio; LR−, negative likelihood ratio.

### Internal correlation among CBC derivative indicators (exploratory analysis)

To elucidate the intrinsic relationships among the six CBC-derived inflammatory markers, we conducted pairwise Spearman correlation analyses. Results revealed statistically significant correlations (*p* < 0.05) in 14 out of 15 marker pairs, forming a tightly interconnected network. The distribution of correlation strength was as follows: three pairs exhibited extremely strong correlations (|*ρ*| ≥ 0.8), nine pairs showed strong correlations (0.6 ≤ |*ρ*| < 0.8), two pairs demonstrated moderate correlations, one pair displayed a weak correlation, and one pair showed no significant correlation. Key correlation patterns are summarized in Table 5.

**Table 5:** Key correlation patterns among CBC-derived inflammatory markers.

Indicator	Indicator 2	Spearman’s ρ	*p* value
NLR	NMLR	0.997	< 0.001
dNLR	MLR	0.087	0.561
MLR	SIRI	0.898	< 0.001
NLR	dNLR	0.799	< 0.001

This table presents Spearman correlation coefficients that are of critical significance for interpreting information redundancy and uniqueness among indicators.

**Figure 3:**
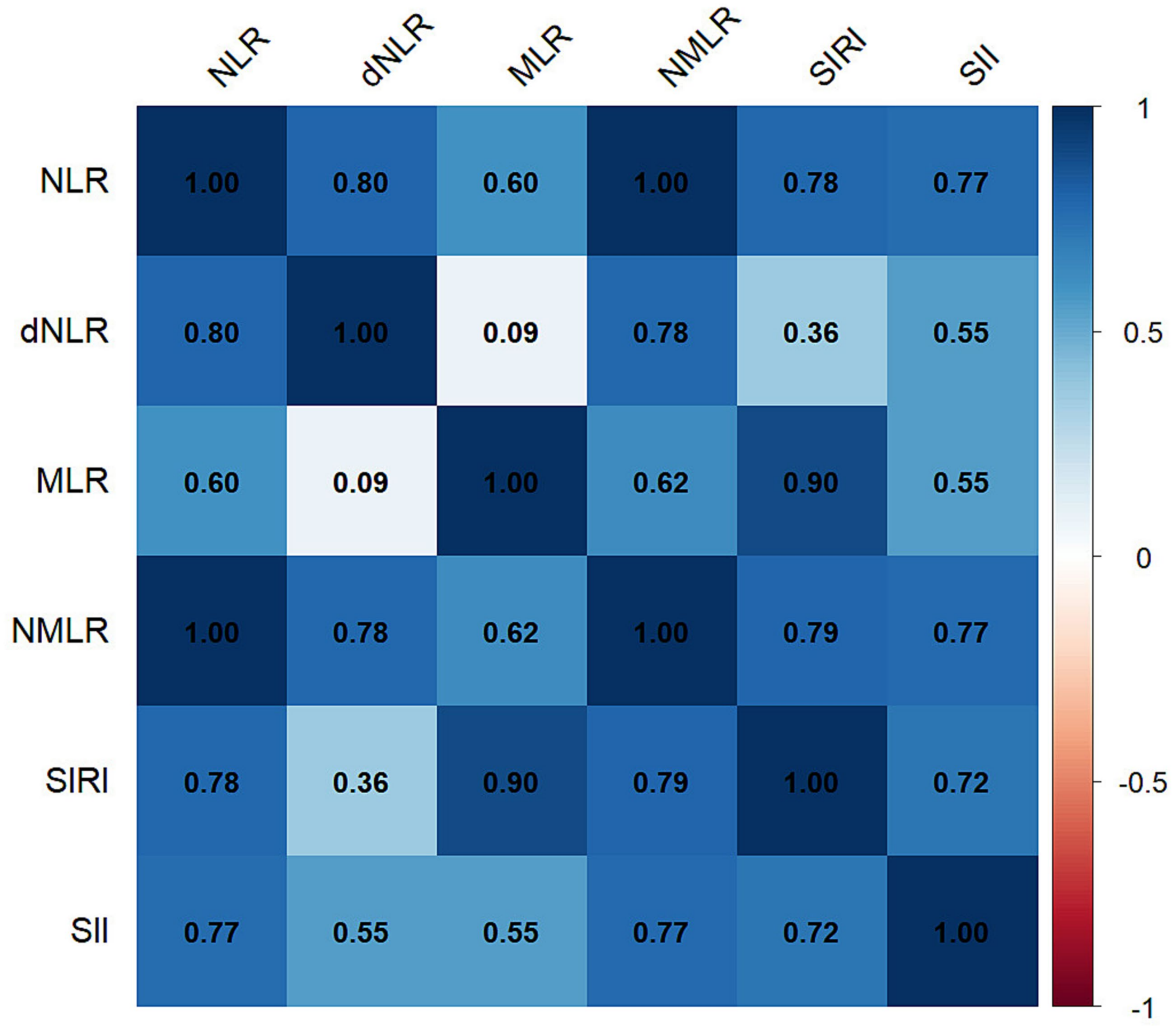
Heatmap of spearman correlations among six CBC-derived inflammatory markers. The values depicted represent Spearman’s rank correlation coefficients (*ρ*). The intensity of color indicates the strength of correlation, typically ranging from light to dark to denote weak to strong associations. It is evident that certain indicators exhibit high correlations (e.g., NMLR with NLR, SIRI with MLR).

The neutrophil-to-lymphocyte ratio (NLR) and the neutrophil-monocyte-to-lymphocyte ratio (NMLR) exhibited near-perfect collinearity (*ρ* = 0.997, *p* < 0.001), suggesting high consistency in their mathematical composition and inflammatory information content. Conversely, the derived neutrophil-to-lymphocyte ratio (dNLR) showed no significant correlation with the monocyte-to-lymphocyte ratio (MLR) (*ρ* = 0.087, *p* = 0.561), and only a weak correlation with the Systemic Inflammatory Response Index (SIRI) (*ρ* = 0.36, *p* = 0.013), suggesting these indices may reflect distinct inflammatory pathways or cellular population dynamics. The MLR and SIRI, however, demonstrated a strong correlation (*ρ* = 0.898, *p* < 0.001). The overall correlation structure, integrating both the strength of correlation coefficients and statistical significance, is visually represented via a heatmap (Figure 3). The complete Spearman correlation coefficient matrix is detailed in Supplementary Table S1.

### Analysis of factors influencing long-term outcomes in patients

To identify factors influencing long-term adverse outcomes (death or entry into permanent dialysis) in patients, we compared clinical indicators between the favorable outcome group (*n* = 42) and the unfavorable outcome group (*n* = 5). As shown in Table 6, traditional renal function indicators exhibited significant differences between the two groups. Patients in the poor-outcome group demonstrated markedly higher levels of creatinine, uric acid, blood urea nitrogen, and serum cystatin C compared to the favourable-outcome group (creatinine: 568.56 vs. 94.76 μmol/L, *p* = 0.004; uric acid: 544.47 vs. 373.00 μmol/L, *p* = 0.020; blood urea nitrogen: 22.36 vs. 7.20 mmol/L, *p* = 0.003; cystatin C: 5.69 vs. 2.15 mg/L, *p* = 0.009). However, no statistically significant differences were observed between the two groups for any CBC-derived inflammatory markers (including NLR, dNLR, MLR, NMLR, SIRI, and SII) (all *p* > 0.05). In summary, within this cohort, traditional markers of renal dysfunction, rather than acute systemic inflammatory indicators, were significantly associated with long-term adverse outcomes in patients.

**Table 6:** Univariate comparison of factors between patients with favorable and poor long-term outcomes.

Variable	Favorable outcome (*n* = 42)	Unfavorable outcome (*n* = 5)	*U*	*p*
Creatinine (μmol/L)	94.76 (70.95, 205.45)	568.56 (519.90, 617.22)	2.700	**0.004**
Uric acid (μmol/L)	373.00 (245.64, 416.50)	544.47 (434.44, 654.50)	2.273	**0.020**
Blood urea nitrogen (mmol/L)	7.20 (5.93, 15.78)	22.36 (19.28, 25.43)	2.806	**0.003**
Cystatin C (mg/L)	2.15 (1.02, 2.73)	5.69 (4.01, 7.37)	2.456	**0.009**
NLR	8.52 (4.55, 18.91)	16.11 (7.73, 51.73)	1.139	0.271
dNLR	0.92 (0.89, 0.95)	0.94 (0.94, 0.99)	2.052	0.152
MLR	0.56 (0.25, 0.96)	0.93 (0.51, 1.42)	1.363	0.179
NMLR	9.42 (4.82, 19.64)	17.13 (8.34, 53.01)	1.104	0.287
SIRI	5.05 (1.31, 11.61)	12.64 (6.11, 16.84)	1.070	0.303
SII	1,957.20 (955.34, 3,703.85)	744.22 (313.68, 11,352.68)	−1.035	0.319

The long-term outcome was defined as death or progression to permanent dialysis after discharge. The favorable outcome group did not experience these events. All continuous variables are presented as median (25th percentile, 75th percentile). Due to the small sample size and non-normal distribution of data, between-group comparisons were performed using the Mann–Whitney *U* test, with the *U* statistic and exact *p*-value reported.

Bold *p*-values (*p* < 0.05) indicate statistically significant differences.

## Discussion

This study presents a systematic investigation of the clinical significance of an inflammatory marker profile from complete blood counts in emphysematous pyelonephritis (EPN). Core findings reveal a critical differentiation phenomenon with distinct “role specialization.” NLR and NMLR strongly correlate with the development of sepsis. dNLR corresponds significantly with radiological anatomical severity, as measured by Huang’s CT grading. Baseline damage severity, shown by conventional renal function measures like creatinine (17–19) and cystatin C, is more important for long-term renal prognosis. This distinction is crucial for understanding how EPN progresses, from local infection to systemic inflammation and, finally, organ outcomes. It raises a key question: why do different indices correspond with unique clinical dimensions? Clarifying these pathways will yield essential insight for targeted therapy and early risk assessment.

### dNLR: the bridge linking local anatomical disruption to systemic inflammatory burden

This study found that dNLR is independently associated with Huang’s CT staging, with its levels increasing progressively across higher staging. This establishes a measurable haematological relationship between “systemic response” and “localized disease.” The calculation method for dNLR [neutrophil count / (total white blood cell count - lymphocyte count)] theoretically provides a more specific reflection of the neutrophil burden relative to other white blood cell populations (20). In the progression of EPN, infectious gases diffuse from the renal collecting system into the renal parenchyma and perirenal spaces, signifying more extensive tissue necrosis and increased release of pathogen-associated molecular patterns into the bloodstream (7, 21). Peripheral blood neutrophil counts will rise sharply as a result of this significant stimulation of myeloid cell production in the bone marrow. As a result, the gradual increase in dNLR may be a direct indicator of the degree of local anatomical destruction-driven systemic granulocyte response. This observation has clear clinical implications: even in cases where vital signs are momentarily stable, a raised dNLR should be considered an early “sentinel” of possible severe systemic inflammation in patients with CT-detected high-grade lesions. It calls for increased attention to detail and more aggressive action.

### NLR and NMLR: identifying the ‘switches’ of systemic immune dysregulation

In contrast to dNLR, NLR and NMLR showed stronger correlations with septic conditions; NMLR had the best discriminatory performance (AUC = 0.683) and the greatest effect size (*r* = 0.45). The essence of sepsis lies in the dysregulation of the host immune response triggered by infection, characterized by the coexistence of excessive inflammation and immunosuppression (6, 22). NLR has been identified as a sepsis risk marker (23, 24). In particular, this biomarker has been shown to be a predictor of the development of systemic inflammatory response syndrome (SIRS) following PCNL in urology (25). In this study, we found that NLR and NMLR were closely associated with the occurrence of sepsis in EPN patients, and that the advent of sepsis and its consequences are the main causes of death in EPN patients (26). Research indicates that 70% of EPN patients develop severe systemic inflammatory response syndrome (SIRS). Among these, 52.6% require admission to the intensive care unit (ICU) due to urosepsis, while 47.4% recover without progressing to sepsis. Neutrophil count signifies an inflammatory storm, while lymphopenia indicates immune paralysis (27).

The third internationally accepted definition of sepsis and septic shock is life-threatening organ dysfunction induced by a dysregulated host response to infection, characterized by the development of dysfunction in any organ distant from the source of infection (5, 28, 29). Septic shock is a form of sepsis characterized by severe underlying circulatory and cellular metabolic dysfunction that dramatically increases mortality. This high mortality rate may be due to the nature of severe infections, in which pathogens can directly penetrate the circulatory system or indirectly produce toxins, resulting in systemic toxicity and tissue hypoperfusion-both of which are associated with multiple organ dysfunction and failure (30). We chose the qSOFA score over the SIRS criteria because previous research has shown that the qSOFA score has a greater specificity for sepsis. Specifically, for patients at risk of uremic sepsis caused by urinary system abnormalities, the qSOFA appears to be superior to the SIRS criteria (31, 32). The NMLR integrates monocyte information within the NLR framework, with the monocyte/macrophage system playing a pivotal role in both the cytokine storm and immune regulation during sepsis. Therefore, NMLR may more comprehensively capture the complex imbalance between excessive activation of the innate immune system (neutrophils, monocytes) and suppression of adaptive immune function (lymphocytes) in the septic state, which may account for its superior discriminatory efficacy. Internal correlation analysis revealed a high collinearity between NLR and NMLR, indicating they fundamentally reflect the same pathophysiological axis, whereas dNLR showed weaker correlations with them. Collectively, these findings suggest that during EPN progression, dNLR primarily functions as a “sentinel of anatomical severity,” while NMLR (representing the NLR axis) acts as a “systemic criticality converter.” Together, they depict disease progression from distinct dimensions, offering potential complementary value.

### Indicator relationships: exposing several aspects of the EPN inflammatory process

This study’s in-depth analysis of internal correlations provides crucial evidence for explaining the aforementioned differentiation phenomenon. Data reveal that NLR exhibits high collinearity with NMLR. This mathematically and biologically explains their consistent performance in associating with sepsis-both fundamentally reflect imbalances within the same pathophysiological axis (the neutrophil/lymphocyte axis). Consequently, in clinical practice, NMLR-possessing a superior effect size-may serve as a representative indicator for this axis. A more revealing finding is that dNLR exhibits only moderate correlation with NLR/NMLR and virtually no correlation with MLR (monocyte-to-lymphocyte ratio) (*ρ* = 0.087). This low correlation strongly suggests that dNLR conveys unique information distinct from conventional cell ratio metrics. It may more sensitively reflect the overall level of granulocyte mobilization directly driven by lesion size, independent of lymphocyte counts. This precisely supports the rationale for its specific association with radiographic anatomical severity.

Thus, we may construct a unified pathophysiological narrative: within the EPN disease spectrum, dNLR serves as a ‘sentinel of disease severity’, with its elevation signaling anatomical progression of infection within localized tissues and the consequent systemic increase in neutrophil burden. Conversely, NLR/NMLR functions as a “clinical criticality converter”; its marked elevation signifies that local infection has breached containment, triggering profound systemic immune-inflammatory dysregulation-the clinical state of septicaemia. These two indicators thus delineate the inflammatory progression of EPN from distinct perspectives.

### The disengagement of inflammatory markers from long-term prognosis and its clinical implications

A clinically significant finding is that all CBC-derived inflammatory markers showed no significant association with long-term hard endpoints (death/permanent dialysis) in patients, whereas traditional renal function markers (creatinine, blood urea nitrogen, cystatin C) demonstrated strong predictive capabilities (17–19). This clearly delineates the distinct clinical applications of different biomarkers. It indicates that the primary determinants of the ultimate renal outcome in EPN patients are the renal reserve capacity present before the acute infectious insult, as well as the severity and reversibility of the acute kidney injury (AKI) episode (7, 33–35). CBC indicators (such as dNLR, NLR, and NMLR) are highly effective “state markers,” sensitively reflecting acute inflammatory storms, whereas traditional renal function indicators are more reliable “prognostic markers,” with abnormalities accumulating to reflect the extent of irreversible parenchymal injury. As a result, clinical management should employ a dual-track assessment strategy: on the one hand, rapidly identify and actively control sepsis-a lethal acute complication-using indicators such as NMLR; on the other hand, prioritize continuous monitoring of renal function trajectories centered on creatinine and cystatin C, as these metrics remain the primary basis for long-term prognosis assessment and renal replacement therapy decisions.

This study has several limitations. First, as a single-center retrospective study, the sample size was limited, particularly with few cases in the Huang II grade and adverse outcome groups. This may affect the stability of subgroup analyses and restrict the ability to conduct reliable multivariate analyses. Second, the cross-sectional design only analyzed baseline data at admission, making it impossible to establish causality or assess the dynamic predictive value of indicators. Future research should involve multicenter, prospective cohort studies to validate these findings in larger samples, with a focus on exploring the following directions: 1. Dynamically monitoring the value of dNLR and NMLR in guiding adjustments to antibiotic treatment intensity and duration; 2. Integrating additional novel immune cell subset assays to elucidate the microscopic mechanisms of EPN immune dysregulation; 3. Integrating imaging grading, inflammatory markers, and renal function indicators to construct comprehensive predictive models for achieving more precise risk stratification and personalized management.

## Conclusion

In conclusion, this study clarifies that CBC-derived inflammatory markers show a clear clinical significance and a distinct associative pattern in EPN. dNLR is used in conjunction with imaging evaluation, and its elevation indicates severe local anatomical destruction and a corresponding systemic inflammatory burden. The best markers for detecting sepsis are NMLR and NLR, which accurately reflect systemic immune-inflammatory imbalance. However, when it comes to predicting long-term prognosis, these acute-phase inflammatory markers cannot take the role of conventional kidney function indicators. These inexpensive, readily available indicators provide clinicians with a practical tool for assessing the severity of EPN and identifying patients at high risk for sepsis. We recommend adopting an integrated assessment strategy in clinical practice, leveraging the unique value of different indicators to separately alert for acute systemic complications and predict long-term renal outcomes. This approach provides practical and cost-effective decision support for the refined diagnosis and treatment of this critical illness.

## Data Availability

The raw data supporting the conclusions of this article will be made available by the authors, without undue reservation.
