# Supplementary material for: dNLR, NLR, and NMLR as differential markers for assessing severity and sepsis in emphysematous pyelonephritis
Source: Front Med (Lausanne). 2026 Jul 15;13:1831260. doi: 10.3389/fmed.2026.1831260 (PMC13416955; doi:10.3389/fmed.2026.1831260)
Supplement: Supplementary file 1 [file Table_1.DOCX]

|  | NLR | dNLR | MLR | NMLR | SIRI | SII |
| --- | --- | --- | --- | --- | --- | --- |
| **NLR** | 1.000 | 0.799 | 0.603 | 0.997 | 0.781 | 0.767 |
| **dNLR** | 0.799 | 1.000 | 0.087 | 0.783 | 0.360 | 0.552 |
| **MLR** | 0.603 | 0.087 | 1.000 | 0.625 | 0.898 | 0.551 |
| **NMLR** | 0.997 | 0.783 | 0.625 | 1.000 | 0.794 | 0.773 |
| **SIRI** | 0.781 | 0.360 | 0.898 | 0.794 | 1.000 | 0.723 |
| **SII** | 0.767 | 0.552 | 0.551 | 0.773 | 0.723 | 1.000 |
| ***Note***: All correlation coefficients (*ρ*) are shown. Coefficients in **bold** highlight the key findings discussed in the main text. | | | | | | |

**Table S1.** Complete matrix of Spearman correlation coefficients among six CBC-derived inflammatory indices.
